# Supplementary material for: Multichannel vectorial holographic display and encryption
Source: Light Sci Appl. 2018 Nov 28;7:95. doi: 10.1038/s41377-018-0091-0 (PMC6258690; doi:10.1038/s41377-018-0091-0)
Supplement: Supplementary file 1 — Supplementary Information [file 41377_2018_91_MOESM1_ESM.docx]

**Supplementary Information**

**Multichannel vectorial holographic display and encryption**

Ruizhe Zhao^1^, Basudeb Sain^2^, Qunshuo Wei^1^, Chengchun Tang^3,4^, Xiaowei Li^5^, Thomas Weiss^6^, Lingling Huang1,2[[1]](#footnote-2), Yongtian Wang1[[2]](#footnote-3), Thomas Zentgraf2[[3]](#footnote-4)

1. *School of Optics and Photonics, Beijing Institute of Technology, Beijing, 100081, China*
2. *Department of Physics, University of Paderborn, Warburger Straße 100, 33098 Paderborn, Germany*
3. *Institute for Quantum Science and Engineering, Shenzhen, 518055, China*
4. *Department of Physics, Southern University of Science and Technology, Shenzhen, 518055, China*
5. *Laser Micro/Nano-Fabrication Laboratory,* *School of Mechanical Engineering, Beijing Institute of Technology,* *Beijing 100081, China*
6. *4th Physics Institute and Research Center SCoPE, University of Stuttgart, Pfaffenwaldring 57, 70569 Stuttgart, Germany*

**A. Derivation of the Jones matrix for** **the multichannel polarization-multiplexed metasurface holograms**

The general relation between the electric ﬁeld of the input (*E^in^*) and the output (*E^out^*) waves at each pixel can be expressed using the Jones matrix. The Jones matrix can be treated as a unitary and symmetric matrix. Therefore, we obtain the following equation^1^:

$T_{xy}=T_{yx}$ and $T_{yy}=-\exp(2i\angle T_{yx})T_{xx}^{*}$ (S1)

where $T_{xx}^{*}$ represents the complex conjugation of *T_xx_*. According to the Jones vector of the input ($E^{in}=\left[\begin{matrix} 1 \\ 0 \end{matrix}\right]$) and the desired output waves () at each pixel and the combination of Eq. 1 (see main text) and Eq. S1, we obtain the Jones matrix of the multichannel polarization-multiplexed metasurface hologram as shown in Eq. 4, which can be used for encoding the phase profile to the metasurface.

**B. Generation algorithm of the hologram**

The flowchart for generating the target phase profiles based on the modified Gerchberg-Saxton algorithm is shown in Figure S1. The original image 1 only participates in the first loop, which is represented by red lines. A feedback function is used for the replacement of the amplitude at the object plane in order to increase the convergence speed and improve the quality of the reconstructed images. After obtaining the desired phase profile *φ_1_* of hologram 1, *φ_1_* is applied to the second iteration which includes image 2 and image 3. The blue and green parts of the flowchart in Fig. S1 represent this iteration. Meanwhile, the relation *φ_3_*=2×*φ_2_*−*φ_1_*+π is used to connect hologram 3 together with hologram 1 and 2. After a sufficient number of iterations, we obtain the optimized phase-only holograms 1, 2 and 3 that enable us to reconstruct independent high-quality holographic images in the far field zone.

**C. The numerical aperture of the holograms**

In the experiment, the objective lens is carefully chosen for collecting the diffracted light from the metasurface hologram sample. The numerical aperture of the hologram can be determined by
 $\frac{l}{N\times p}=\frac{\cos(\frac{\pi}{2}-\alpha)}{\lambda}$ (S2)
 $NA=n\times\sin\alpha$ (S3)

where *N* and *p* represent the number of pixels and pixel size of the metasurface, respectively, and *l* indicates the distance from a point of the original image to its center. The diffraction angle is represented by *α* in Eqs. S2 and S3. For the generation of the holograms, *l_max_* in the original image determines the largest diffraction angle, which results in the minimum required *NA_min_* of the collecting objective lens needed in the experiment. The numerical aperture of our objective lens used in the experiment is 0.6 (*NA_max_* of our designed metasurfaces hologram is 0.45). This guarantees that the entire *k*-space of the hologram can be captured by our imaging system.

**D. The** **feasibility** **of achieving a dynamic holographic display**

In order to demonstrate the feasibility of achieving a dynamic holographic display, we choose three independent original images, which represent a cartoon of a person running, jumping and landing on the ground. The simulated and experimental results of this sample under illuminating with linearly and circularly polarized beams are shown in Figure S2. Three different independent images and all the combinations of these images (twelve channels and seven different combinations) can be obtained by combining different input/output polarizations. By continuously changing the polarization of the input and output light in the correct order, we can observe these three images to appear and reconstruct successively. Such dynamic image projection based on different combinations of polarization states may open a new frontier for applications related to dynamic holographic displays (for example see the supplementary video S4).

**E. Diffraction efficiency of the metasurface holograms**

The key for achieving multichannel polarization multiplexing relies on the birefringent and rotational properties of simple nanofin structures with a rectangular cross-section. The statistical results of the number of different kinds (different cross-sections and orientation angles) of nanofins that are contained in our designed metasurfaces holograms are shown in Figure S3. In the 3D coordinate system [*N*(*L,w,θ*)], the solid spheres represent the different kinds of nanofins and the colors indicate the amount of each kind of nanofins. Two projections of these distributions are shown in the 3D coordinate system in order to describe the distribution of nanofins with different orientation angles and cross-sections. By using a rigorous coupled wave analysis method, we obtained the transmission coefficients (*t_xx_*, *t_xy_*, *t_yx_,* and *t_yy_*) of different kinds of nanofins for the purpose of calculating the theoretical transmission efficiencies of the metasurface samples. Take the “meta” sample as an example, the calculated theoretical diffraction efficiencies *T_xx_*, *T_xy_*, *T_yx_,* and *T_yy_* are 42.88%, 37.2%, 37.21%, and 43.01%, respectively. The results measured by an FTIR spectrometer for the four samples are shown in Figure S4, with satisfactory efficiencies at the working wavelength of 800 nm. The difference between the theoretical and experimental results might be caused by imperfection of fabrication and material loss.

**F. Broadband properties of the metasurface holograms**

By changing the wavelength of the incident light from the near-infrared region to visible light, we find that the reconstructed images can still be observed with high fidelity. The results for the metasurface hologram sample with the tiger/snowman/teapot/cup are shown in Figure S5. As the magnifying ratio *M* of the reconstructed images is defined by $M\text{=}\frac{\lambda f}{p^{2}N}$, where *f* and *p* represent the focal length of used microscope objective lens (that is used in the experiment) and the pixel size of metasurface, respectively. *N* represents the pixel number for one spatial dimension of the hologram. Therefore, the sizes of the reconstructed images decrease for shorter wavelengths. When the wavelength of the incident light is changed over a relatively broad range between 600-800 nm, the variation of the corresponding refractive index of amorphous silicon is quite small. We further calculated the phase shift *φ’_x_(λ)* and *φ’_y_(λ)* at different wavelengths and substitute those values to the corresponding pixels of metasurface hologram for the investigation of such a broadband phenomenon (Figure S7). While for the multichannel vectorial hologram samples with the rotation of the nanofins, we can acquire the renewed *T* matrix based on Eq. 2 once the geometric parameters of the nanofin at each pixel are fixed. The Fourier transform is applied in order to reconstruct the images in the broadband spectrum (Figure S5 and S6). We noted for the rotated nanofins, the broadband range is narrower (ranging from 835 to 690 nm). The simulated results are consistent with the experimental results. The broadband response arises from the robustness of our silicon metasurface holograms and the fact that holograms generated by the GS algorithm are wavelength independent. The reconstructed images can be observed with acceptable quality even apart from our design wavelength of 800 nm by using dielectric metasurfaces. Furthermore, according to Fig. S3, most sizes of the nanofins are very similar as can be seen from the localization region (white and red spheres), while the phase difference distribution for nanofins located in this region is nearly constant (white dashed box in Fig. S7). Noted that the intensities of the reconstructed images decrease and the conjugated images will be observed when the wavelength of incident light is moving towards 600 nm in both the simulation and experiment.

**G. Recorded videos by continuously rotating a linear polarizer**

Video S1: Two-channel polarization and angle-multiplexed hologram (“cartoon tiger”, “cartoon snowman”, “teapot”, “cup”).

Video S2: Multichannel polarization-multiplexed holograms (“holography”, “meta”, “surface”).

Video S3: Multichannel polarization-multiplexed holograms (“dice”).

Video S4: Multichannel polarization-multiplexed holograms (“cartoon person”)

All videos are captured by a regular CCD camera and the first and second white arrows represent the polarization combination of incident/transmit, respectively.


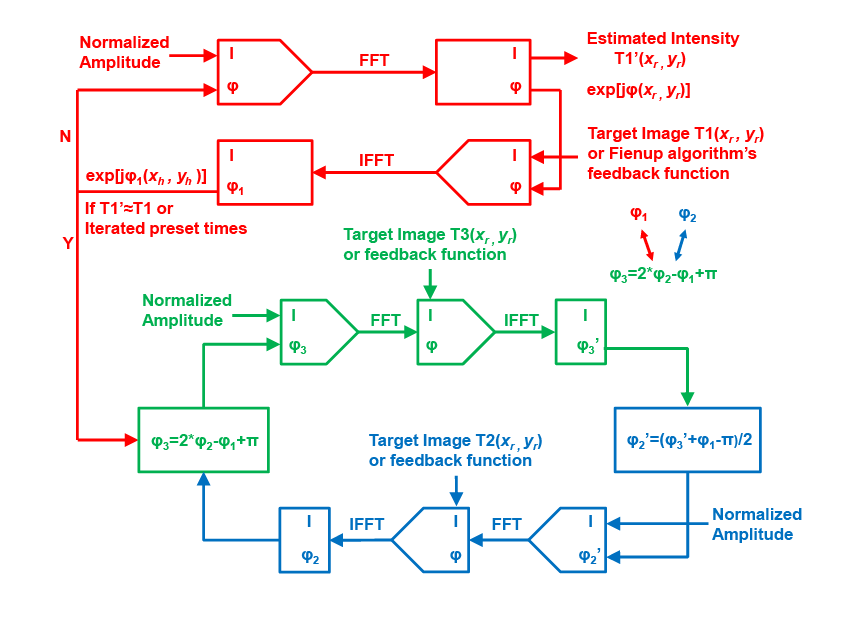


**Fig. S1** The flow chart of the modified Gerchberg-Saxton algorithm for correlating all the required phase distribution. The red, blue and green parts of the chart represent the processes involved by the original images 1, 2, and 3, which are related to phase profile of *φ_1_*, *φ_2_,* and *φ_3_*, respectively. “FFT” and “IFFT” represent the Fourier transform and inverse Fourier transform.

**
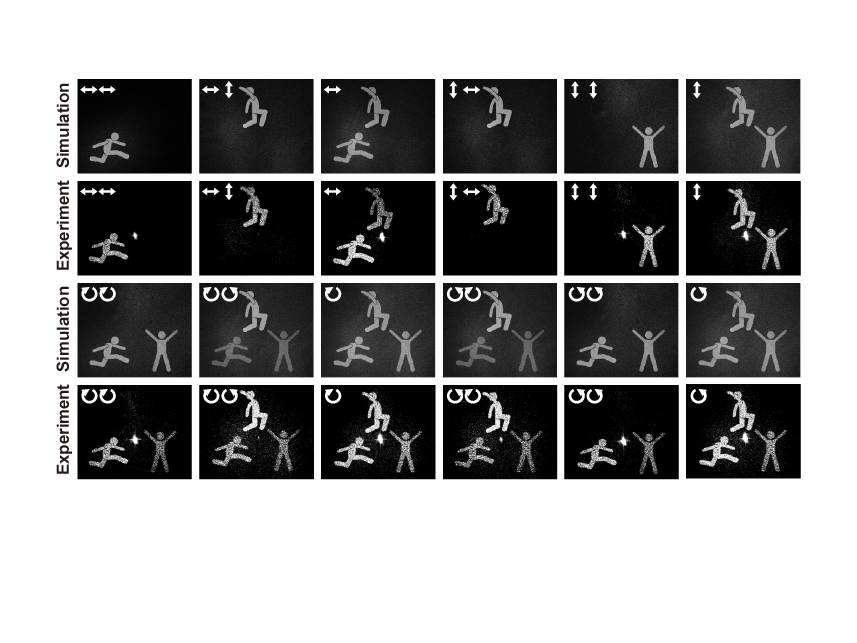
**

**Fig. S2** Simulated and experimental results for the multichannel polarization-multiplexed hologram showing a cartoon person under illumination of linearly and circularly polarized light. The two white arrows at the corners indicate the input (first arrow) and the output (second arrow) polarizations of light. The dynamical behavior for a continuous rotation of the polarizer is shown in the video S4.


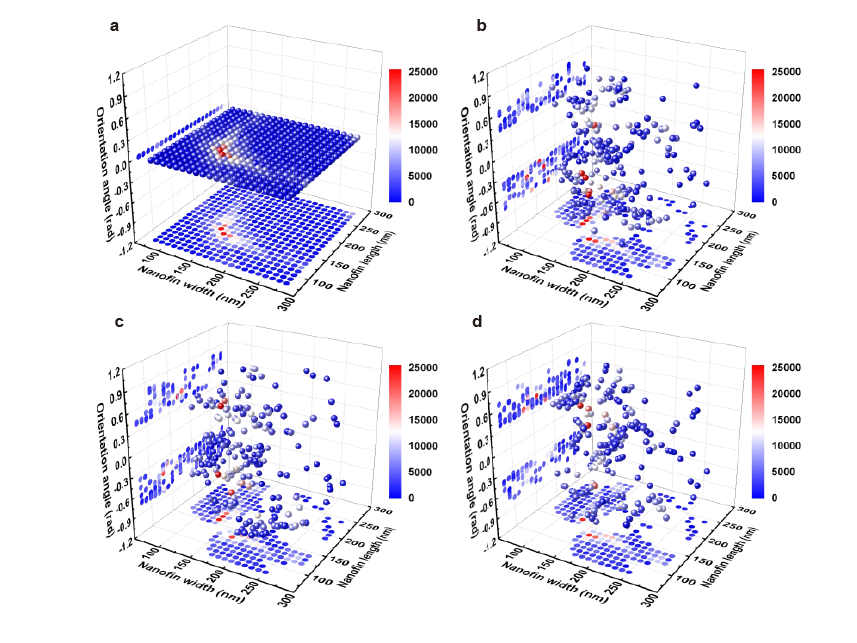


**Fig. S3** Statistical results of the number of each nanofin (different cross-sections and orientation angles) that are contained in our designed metasurface holograms. The solid spheres represent a different cross-section of the nanofins and the colors of spheres indicate the number of these nanofins with the particular cross-section. **a** Two-channel polarization- and angle-multiplexed hologram (“cartoon tiger”, “cartoon snowman”, “teapot”, “cup”) **b** Multichannel polarization-multiplexed hologram (“holography”, “meta”, “surface”) **c** Multichannel polarization-multiplexed hologram (“dice”) **d** Multichannel polarization-multiplexed hologram (“cartoon person”).


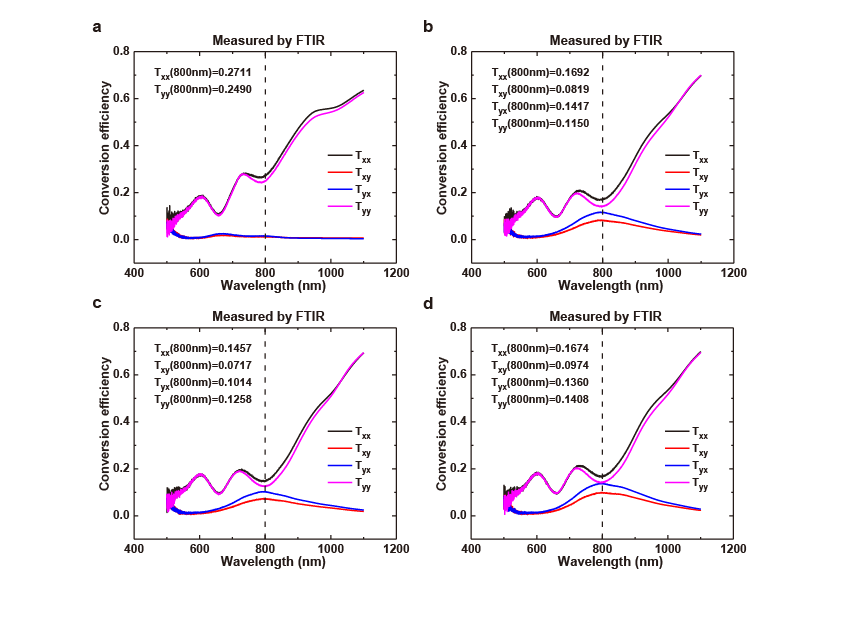


**Fig. S4** Polarization conversion efficiencies of fabricated metasurfaces samples measured by FTIR. *T_ij_* represents the conversion efficiency when the incident light is *j* linearly polarized beams and the transmission is *i* linearly polarized. **a** Two channels polarization and angle multiplexed hologram (“cartoon tiger”, “cartoon snowman”, teapot, cup) **b** Multichannel polarization multiplexed holograms (“holography”, “meta”, “surface”) **c** Multichannel polarization multiplexed holograms (“dice”) **d** Multichannel polarization multiplexed holograms (“cartoon person”).


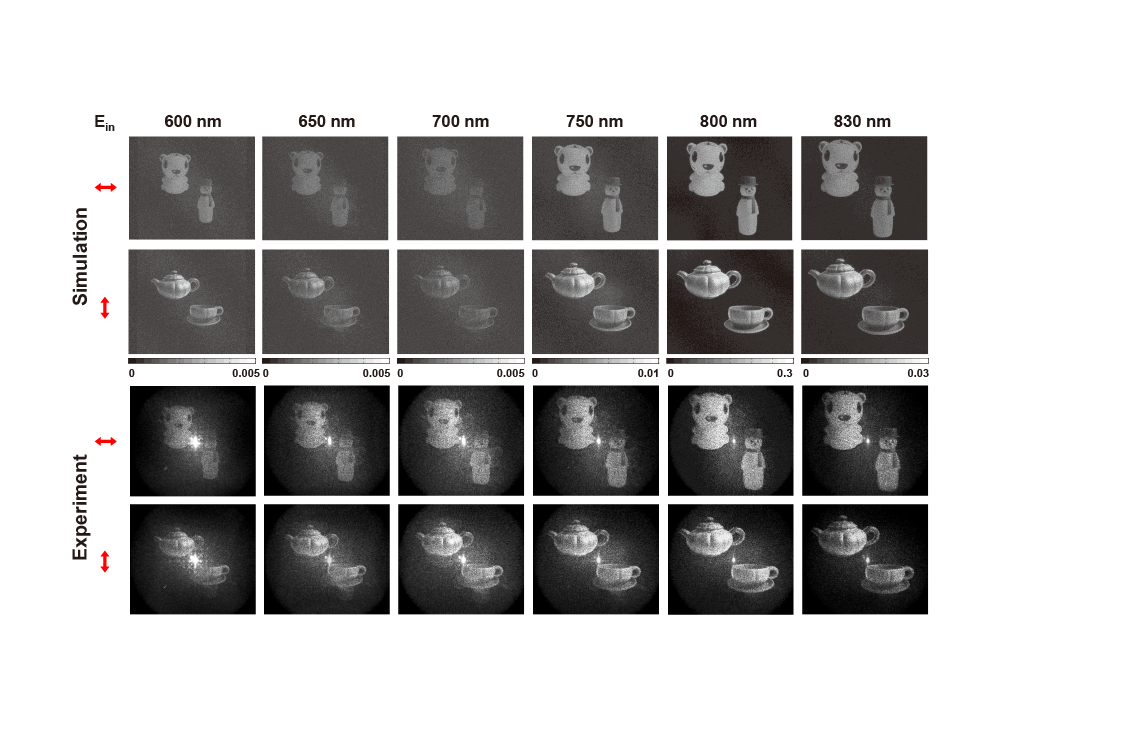


**Fig. S5** Broadband properties of our designed metasurface holograms. The red arrows indicate the polarization of incident light. In the simulation, we replace the phase shift at different incident wavelengths generated by the metasurfaces for the reconstruction of the holographic image. For the experiment, we utilize a tunable laser for the investigation. Both the simulation and the experiment corresponding to each other.

**
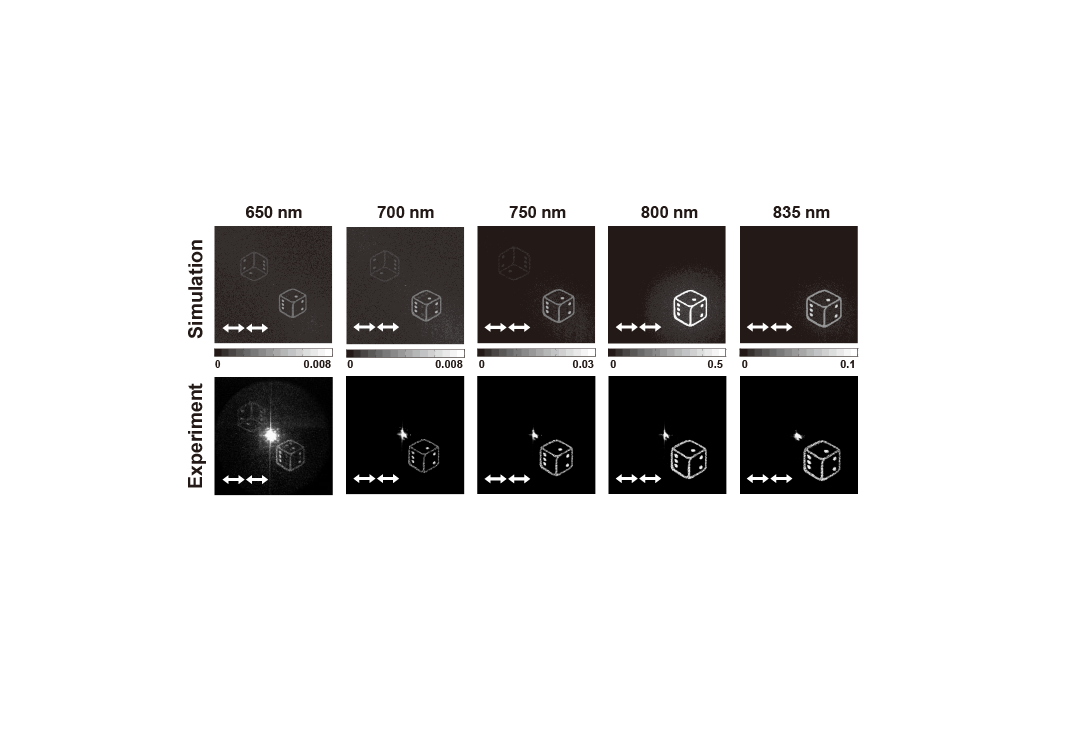
**

**Fig. S6** Broadband effect for the multichannel vectorial hologram with the rotation of the azimuthal angles. The reconstruction spectrum ranges from 650 to 835 nm. For the experiment, we utilize a tunable laser for the investigation.


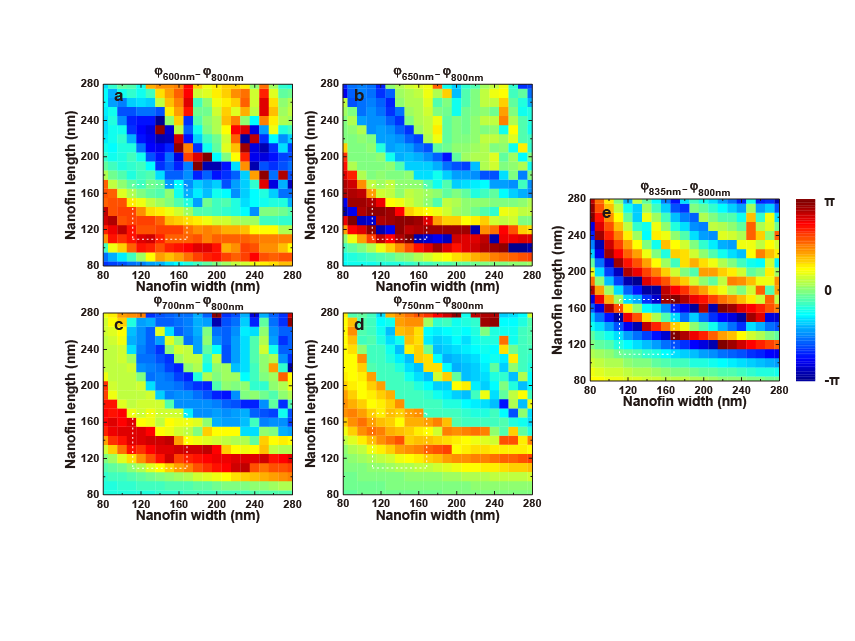


**Fig. S7** **a-e** Phase shift of *t_xx_* at different incident wavelengths compared to the working wavelength of 800 nm, which are used to reconstruct the hologram theoretically and help to explain the broadband effect of such dielectric metasurfaces.

**Reference**

1. Arbabi, A., Horie, Y., Bagheri, M. & Faraon, A. Dielectric metasurfaces for complete control of phase and polarization with subwavelength spatial resolution and high transmission. *Nat. Nanotechnol.* **10**, 937-943 (2015).

1. Email: huanglingling@bit.edu.cn [↑](#footnote-ref-2)
2. Email: wyt@bit.edu.cn [↑](#footnote-ref-3)
3. Email: thomas.zentgraf@upb.de [↑](#footnote-ref-4)
